# A pBBR1‐based vector with IncP group plasmid compatibility for *Methylorubrum extorquens*

**DOI:** 10.1002/mbo3.1325

**Published:** 2022-10-04

**Authors:** Laura Pöschel, Elisabeth Gehr, Markus Buchhaupt

**Affiliations:** ^1^ DECHEMA‐Forschungsinstitut Microbial Biotechnology Frankfurt am Main Germany; ^2^ Department of Life Sciences Goethe University Frankfurt Frankfurt am Main Germany

**Keywords:** cotransformation, expression system, *Methylorubrum extorquens* AM1, plasmid, plasmid copy number, Rep gene

## Abstract

Plasmids are one of the most important genetic tools for basic research and biotechnology, as they enable rapid genetic manipulation. Here we present a novel pBBR1‐based plasmid for *Methylorubrum extorquens*, a model methylotroph that is used for the development of C1‐based microbial cell factories. To develop a vector with compatibility to the so far mainly used pCM plasmid system, we transferred the pBBR1‐based plasmid pMiS1, which showed an extremely low transformation rate and caused a strong growth defect. Isolation of a suppressor mutant with improved growth led to the isolation of the variant pMis1_1B. Its higher transformation rate and less pronounced growth defect phenotype could be shown to be the result of a mutation in the promotor region of the *rep* gene. Moreover, cotransformation of pMis1_1B and pCM160 was possible, but the resulting transformants showed stronger growth defects in comparison with a single pMis1_1B transformant. Surprisingly, cotransformants carrying pCM160 and a pMis1_1B derivative containing a *mCherry* reporter construct showed higher fluorescence levels than strains containing only the pMis1_1B‐based reporter plasmids or a corresponding pCM160 derivative. Relative plasmid copy number determination experiments confirmed our hypothesis of an increased copy number of pMis1_1B in the strain carrying both plasmids. Despite the slight metabolic burden caused by pMis1_1B, the plasmid strongly expands the genetic toolbox for *M. extorquens*.

## Graphical abstract

The pBBR1 derivative pMis1_1B, which was characterized in this study, represents a novel plasmid for *Methylorubrum extorquens*, which is compatible with the widely used pCM system. The growth defect caused by the plasmid was found to be enhanced by the presence of a pCM plasmid, but cotransformation could be demonstrated to be possible.

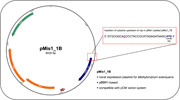

## 1 INTRODUCTION

*Methylorubrum extorquens* has great potential to become a universal production strain for the C1‐based bioeconomy (Chen & Lan, 2020; Ochsner et al., 2014; Zhang et al., 2019). In the last decade, several *M. extorquens* strains with heterologously expressed metabolic pathways were described for the biotechnological production of various products including 1‐butanol, 3‐hydroxypropionic acid, mono‐ and dicarboxylic acids, mevalonate or α‐humulene (Hu & Lidstrom, 2014; Liang et al., 2017; Lim et al., 2019; Schada von Borzyskowski et al., 2018; Sonntag et al., 2014, Sonntag, Kroner, et al., 2015; Sonntag, Müller, et al., 2015; Yang et al., 2017). The development of production strains, however, is limited by the organism's restricted genetic accessibility. Although the bacterium has served as a model organism for methylotrophy for many decades and is therefore well described, only a few genetic tools have been developed. Plasmids are a key tool for the biotechnological applicability of a strain. The simultaneous use of two independent plasmids provides experimental flexibility and simplifies screenings and production strain developments. The compatibility of two or more plasmids in a single bacterial cell requires not only different selection markers but, more importantly, different origins of replication and partitioning systems (del Solar et al., 1998; Novick, 1987). As part of their characterization, plasmids are divided into Inc groups: plasmids from the same group are usually incompatible.

For *M. extorquens* AM1, besides a recently described set of mini chromosomes (Carrillo et al., 2019), derivatives of the pCM plasmid system (Marx & Lidstrom, 2001) were used almost exclusively as episomal vectors for heterologous gene expression. The expression vectors pCM80 (tc^R^) and pCM160 (kan^R^) are based on pDN19, a small IncP vector (Marx & Lidstrom, 2001). The authors of the respective study isolated the derivative pDN19X from *M. extorquens* AM1 transformed with pDN19. This derivative could be efficiently maintained and retransferred in *M. extorquens* AM1 and was used as a basis for the development of the pCM system (Marx & Lidstrom, 2001). In their work, the authors also showed that pBBR1MCS‐2, a vector derived from *Bordetella bronchiseptica* pBBR1, could be transferred into *M. extorquens* with very low transformation efficiency and a significantly reduced transformant growth rate. Since this vector is known to be compatible with IncP group plasmids (Antoine & Locht, 1992; Kovach et al., 1994, 1995), we chose it as a starting point for the development of a plasmid with compatibility with the pCM vector system. The GC content of 64.6% seems to be applicable to *M. extorquens* AM1 (overall GC content of 68.5% [Vuilleumier et al., 2009]) and its diverse usability as a broad range vector can be also useful for certain applications. Here, we describe a pBBR1‐derived plasmid with a mutation upstream of the *rep* gene that confers suitability for cotransformation with established pCM plasmids.

## 2 MATERIALS AND METHODS

### 2.1 Plasmids and bacterial strains

A list of all used plasmids and bacterial strains is given in Table 1. Reporter gene plasmids were constructed by subcloning synthesized DNA fragments (BioCat) into plasmid backbones. Used sequences and restriction enzymes are listed in Table 2. Analysis of sequences and creation of plasmid map was done with SnapGene (www.snapgene.com). Transformation of *M. extorquens* AM1 with plasmid DNA was performed as previously described (Toyama et al., 1998). If not stated differently, 100 ng of plasmid DNA was used for transformations.

**Table 1:** Plasmids and bacterial strains used in this study

Name	Relevant features	Reference
**Bacterial strains**		
*Escherichia coli* DH5α	F^–^ φ80*lac*ZΔM15, Δ(*lac*ZYA‐*arg*F)U169, *rec*A1, *end*A1, *hsd*R17 (r_K_^–^, m_K_^+^) *pho*A, *sup*E44, λ^–^, *thi*‐1 *gyr*A96 *rel*A1	ATCC
*Methylorubrum extorquens* AM1	Cm^R^, gram‐negative, facultatively methylotrophic, obligate aerobic α‐proteobacterium	Peel and Quayle (1961)
**Plasmids**		
pCM160	Constitutive expression vector for *M. extorquens*; Kan^R^, pmxaF (IncP)	Marx and Lidstrom (2001)
pCM80	Constitutive expression vector for *M. extorquens*; Tc^R^, pmxaF (IncP)	Marx and Lidstrom (2001)
pMis1	*Pseudomonas putida* expression vector, Kan^R^	Mi et al. (2014)
pMis4	*P. putida* expression vector; pMis1 derivative with mutated *rep* gene, Kan^R^	Mi et al. (2016)
pBBR1MCS‐2	broad‐host‐range expression vector, Kan^R^	Kovach et al. (1995)
pMis1_1B	pMis1 derivative, Kan^R^	This study (GenBank OP441404)
pCM160_*mCherry*	pCM160‐based mCherry reporter plasmid	This study
pMis1_1B_P_mxaF__*mCherry*	pMis1_1B based mCherry reporter plasmid, promoter *rha*P_BAD_ was exchanged with P_mxaF_	This study

**Table 2:** Synthetic sequences used for cloning of reporter plasmids. DNA was synthesized by BioCat (Heidelberg, Germany). Underlined nucleotides mark restriction sites for cloning

Plasmids/Sequences	Restriction enzymes used	Backbone
**pMis1_1B_P**_**mxaF**_ **_** ***mCherry***		
GAATTCCCCGCTTGGTCGGGCCGCTTCGCGAGGGCCCGTTGACGACAACGGTGCGATGGGTCCCGGCCCCGGTCAAGACGATGCCAATACGTTGCGACACTACGCCTTGGCACTTTTAGAATTGCCTTATCGTCCTGATAAGAAATGTCCGACCAGCTAAAGACATCGCGTCCAATCAAAGCCTAGAAAATATAGGCGAAGGGACGCTAATAAGTCTTTCATAAGACCGCGCAAATCTAAAAATATCCTTAGATTCACGATGCGGCACTTCGGATGACTTCCGAGCGAGCCTGGAACCTCAGAAAAACGTCTGAGAGATACCGCGGAGACGTCATGGTGAGCAAGGGCGAGGAGGATAACATGGCCATCATCAAGGAGTTCATGCGCTTCAAGGTGCACATGGAGGGCTCCGTGAACGGCCACGAGTTCGAGATCGAGGGCGAGGGCGAGGGCCGCCCCTACGAGGGCACCCAGACCGCCAAGCTGAAGGTGACCAAGGGTGGCCCCCTGCCCTTCGCCTGGGACATCCTGTCCCCTCAGTTCATGTACGGCTCCAAGGCCTACGTGAAGCACCCCGCCGACATCCCCGACTACTTGAAGCTGTCCTTCCCCGAGGGCTTCAAGTGGGAGCGCGTGATGAACTTCGAGGACGGCGGCGTGGTGACCGTGACCCAGGACTCCTCCCTGCAGGACGGCGAGTTCATCTACAAGGTGAAGCTGCGCGGCACCAACTTCCCCTCCGACGGCCCCGTAATGCAGAAGAAGACCATGGGCTGGGAGGCCTCCTCCGAGCGGATGTACCCCGAGGACGGCGCCCTGAAGGGCGAGATCAAGCAGAGGCTGAAGCTGAAGGACGGCGGCCACTACGACGCTGAGGTCAAGACCACCTACAAGGCCAAGAAGCCCGTGCAGCTGCCCGGCGCCTACAACGTCAACATCAAGTTGGACATCACCTCCCACAACGAGGACTACACCATCGTGGAACAGTACGAACGCGCCGAGGGCCGCCACTCCACCGGCGGCATGGACGAGCTGTACAAGTAATCTAGA	*Eco*RI + *Xba*I	pMis1_1B
**pCM160_** ***mCherry***		
GCATGCATGGTGAGCAAGGGCGAGGAGGATAACATGGCCATCATCAAGGAGTTCATGCGCTTCAAGGTGCACATGGAGGGCTCCGTGAACGGCCACGAGTTCGAGATCGAGGGCGAGGGCGAGGGCCGCCCCTACGAGGGCACCCAGACCGCCAAGCTGAAGGTGACCAAGGGTGGCCCCCTGCCCTTCGCCTGGGACATCCTGTCCCCTCAGTTCATGTACGGCTCCAAGGCCTACGTGAAGCACCCCGCCGACATCCCCGACTACTTGAAGCTGTCCTTCCCCGAGGGCTTCAAGTGGGAGCGCGTGATGAACTTCGAGGACGGCGGCGTGGTGACCGTGACCCAGGACTCCTCCCTGCAGGACGGCGAGTTCATCTACAAGGTGAAGCTGCGCGGCACCAACTTCCCCTCCGACGGCCCCGTAATGCAGAAGAAGACCATGGGCTGGGAGGCCTCCTCCGAGCGGATGTACCCCGAGGACGGCGCCCTGAAGGGCGAGATCAAGCAGAGGCTGAAGCTGAAGGACGGCGGCCACTACGACGCTGAGGTCAAGACCACCTACAAGGCCAAGAAGCCCGTGCAGCTGCCCGGCGCCTACAACGTCAACATCAAGTTGGACATCACCTCCCACAACGAGGACTACACCATCGTGGAACAGTACGAACGCGCCGAGGGCCGCCACTCCACCGGCGGCATGGACGAGCTGTACAAGTAAGCATGC	*Sph*I	pCM160

### 2.2 Media and culture conditions

*Escherichia coli* DH5α (NEB) was used for plasmid amplification and cloning. *E. coli* cultures were grown in LB (lysogeny broth) medium (Bertani, 1951). *M. extorquens* AM1 (Peel & Quayle, 1961) cultivations were performed in a liquid minimal medium (Peyraud et al., 2009) with 123 mM methanol and a final concentration of 12.6 µM CoCl2 (Kiefer et al., 2009; Sonntag et al., 2014). Solid medium contained 1.5% [w/v] agar‐agar. Antibiotics were used in concentrations of 50 µg/ml for kanamycin or 10 µg/ml for tetracycline hydrochloride. All media components were purchased from Carl Roth or Merck. *M. extorquens* AM1 main cultures were inoculated to an OD_600_ value of 0.1 with precultures grown for 48 h at 30°C and 180 rpm on a rotary shaker.

### 2.3 Growth monitoring and fluorescence assays

For the high‐resolution monitoring of growth and mCherry fluorescence signals, *M. extorquens* AM1 cultures were grown in a BioLector® microbioreactor system (m2p‐labs GmbH). Main cultures were grown in Flowerplate® wells at 30°C, 1000 rpm, and 85% humidity. The final cultivation volume was 1 ml. The level of mCherry reporter protein was measured via its fluorescence signal at 580/610 nm [ex/em]. Growth was monitored via scattered light signals.

### 2.4 Determination of relative plasmid copy numbers

The relative plasmid copy number (PCN) of pMis1_1B_*mCherry* was determined by comparing plasmid‐specific real‐time PCR fluorescence signals in strains containing two plasmids (=sample) to strains containing one plasmid (=control). The main cultures of respective strains were inoculated and cultivated as described above. Cells were harvested in the mid to late exponential growth phase after 45 h of cultivation. DNA was isolated with a QIAamp® DNA Mini kit (Qiagen) following the manufacturer's instructions for bacterial cells. Real‐time PCR primers pairs (Table 3) were designed for amplification of a specific region of the resistance markers on the plasmids (=target) as well as for amplification of a genomic reference sequence (=Ref), all yielding amplicons of 140–143 nucleotides in length. Real‐time PCR experiments were performed using QuantiTect® SYBR® Green PCR Kit in a PikoReal™ System (Thermo Scientific) according to the manufacturer's instructions. The following PCR protocol was used: 15 min at 95°C followed by 45 cycles of 94°C/15 s, 50°C/30 s, and 72°C/30 s. The specificity of primers was confirmed by melting curves and gel electrophoresis. The dynamic range of reaction was validated by testing a tenfold serial dilution of DNA template ranging from 50 ng to 5 pg. The efficiency (E) was subsequently calculated with Equation (1) (Higuchi et al., 1993; Rasmussen, 2001).

(1) $$E=10^{-1/\mathrm{slope}}$$

**Table 3:** Primers used for real‐time polymerase chain reactions

Name	Sequence	Target of amplification
LPoe144_qPCR_fw_ref	GATCAGCGTGACGTACTG	Parts of gene *kgtP* (genomic DNA = “Ref”)
LPoe145_qPCR_rev_ref	CCGGTTCTTCTCGTGATC	
LPoe150_qPCR_fw_pMis	CGAGGATCTCGTCGTGACC	Parts of Kan^R^ cassette on pMis (plasmid DNA = “target”)
LPoe151_qPCR_rev_pMis	TATCACGGGTAGCCAACGC	

The amount of starting material for the final experiments was 500 pg of template DNA. All reactions were performed in biological triplicates. The relative ratio (R) was calculated with the Pfaffl efficiency‐corrected model with averaged controls (Equation 2): (Pfaffl, 2004).

(2) $$R=\frac{{{(E}_{\mathrm{target}})}^{∆C_{P\mathrm{target}}\left(\mathrm{MEAN}\mathrm{control}-\mathrm{sample}\right)}}{{{(E}_{\mathrm{Ref}})}^{∆C_{P\mathrm{Ref}}\left(\mathrm{MEAN}\mathrm{control}-\mathrm{sample}\right)}}$$

## 3 RESULTS AND DISCUSSION

### 3.1 Increased transformation efficiency of pMis variant pMis1_1B

**Figure 1:**
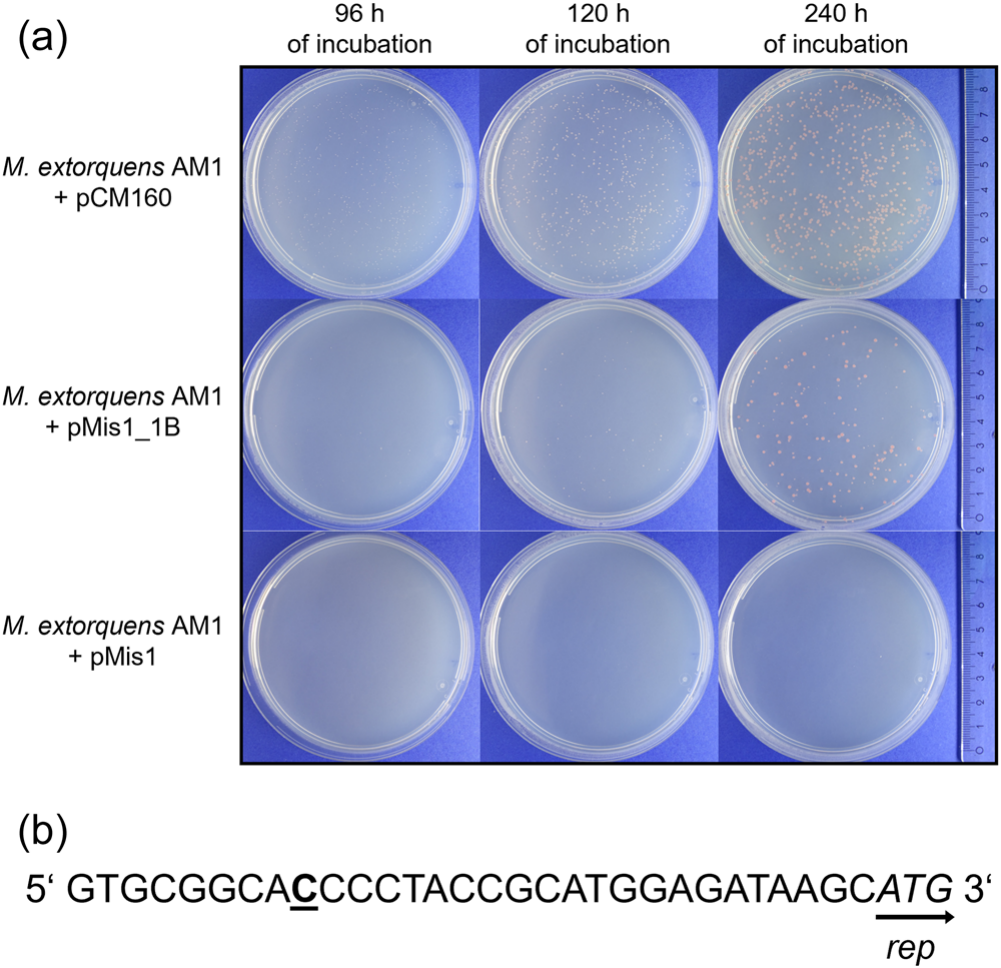
Growth of *Methylorubrum extorquens* AM1 after transformation with pCM160 or pBBR derivatives and DNA sequence differences between the used pBBR derivatives pMis1 and pMis1_1B. (a) Transformation plates after transformation of *M. extorquens* AM1 with 30 fmol of pMis1, pMis1_1B, or pCM160. Transformation plates were photographed after 96, 120, and 240 h of incubation. (b) Mutation in the sequence of pMis1_1B. The inserted nucleotide (cytosine) is located 22 bp upstream of the *rep* gene, whose start codon is indicated by italic letters.

We tested different variants of pBBR1 (Antoine & Locht, 1992) for transformability in *M. extorquens* AM1, as pBBR1MCS‐2 was transferred into *M. extorquens* AM1 already, albeit with very low efficiency (Marx & Lidstrom, 2001). Plasmids pMis1 and pMis4 are derivatives of pBBR1MCS‐2 carrying a rhamnose‐inducible promoter (Mi et al., 2016). In pMis4, a mutation in the *rep* gene leads to a G159S modification in the Rep protein. This mutation prevented the plasmid burden caused by pMis1 in *Pseudomonas putida* (Mi et al., 2016 *)*. The named plasmids and a pCM160 control were transferred into *M. extorquens* AM1, and an aliquot of 100 µl of the transformation mix was spread on selective agar plates. Although transformation of *M. extorquens* AM1 with pCM160 yielded almost a lawn of colonies after 96 h of incubation, other plasmids yielded a low number of transformants after 192 h of incubation: On the pBBR1MCS‐2 transformation plate, only 2 very small colonies were visible, indicating a severe growth defect of the respective transformants. Transformation with pMis1 yielded in a sole, big colony, and transformation with pMis4 in a small number of big colonies. The respective plasmids of these colonies were isolated and 30 fmol of pDNA was retransformed into *M. extorquens* AM1 along with pCM160 as a control. Only the plasmid isolated from the pMis1 transformant showed a clear increase in transformation efficiency compared to pMis1 and was named pMis1_1B (Figure 1a). Colonies of pMis1_1B transformants were clearly visible after 96 h of incubation, with inconsistent colony size, whereas colonies of pMis1 transformants were not visible even after 240 h of cultivation. Complete sequencing of pMis1_1B revealed an insertion of one cytosine residue 22 bp upstream of the *rep* gene (Figure 1b). This insertion upstream of the rep region has likely altered the plasmid copy number (PCN). For pBBR1‐based plasmids, the Rep protein is known to play a crucial role in PCN control by binding to the origin of replication (ori) (Antoine & Locht, 1992; del Solar et al., 1998). Consequently, modifications in the *rep* gene region have been shown to influence the PCN (Mi et al., 2016; Tao et al., 2005; Wadood et al., 1997). Interestingly, the currently used pCM system is based on a similar experiment with the plasmid pDN19 (Marx & Lidstrom, 2001). pDN19 was transferred into *M. extorquens* AM1 with low efficiency, but a mutated derivative (pDN19X) could be isolated from a transformant. pDN19X was efficiently maintained and showed enhanced transformation efficiency. The described single‐point mutation in *traJ* led to changes in transformability even though all *traJ* functions were provided by helper plasmids. The authors, therefore, speculated that transformability was not enhanced by changes in TraJ but by a change in PCN. This confirms that the PCN is an extremely important factor for *M. extorquens* plasmids.

### 3.2 Compatibility of pMis1_1B with the established pCM plasmids

**Figure 2:**
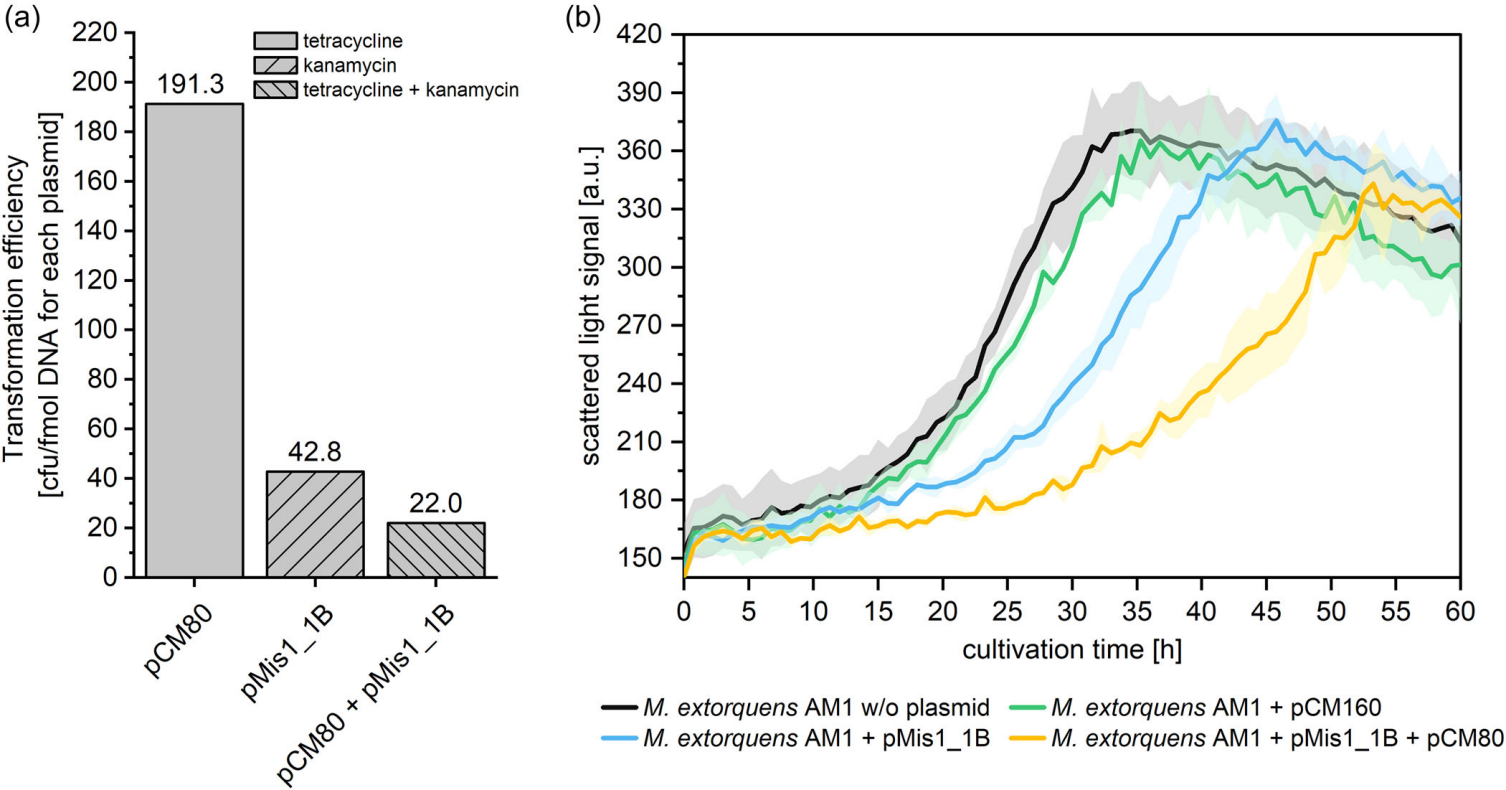
Compatibility of pMis1_1B and pCM80. (a) Transformation efficiencies of pCM80 and pMis1_1B for single and double transformations. Transformation mixtures contained 30 fmol plasmid DNA for single plasmid transformations and 30 fmol plasmid DNA of each plasmid in double transformations. Transformation mixtures were plated on a solid medium with antibiotics for the corresponding plasmid(s). Antibiotics used are indicated by the bar pattern. Colonies of single transformants were counted after 144 h of growth, while colonies of cotransformants were counted after 192h of growth. (b) Growth of *Methylorubrum extorquens* AM1 in a microbioreactor system containing no plasmid, pCM160, pMis1_1B, or pMis1_1B and pCM80 in combination, respectively. Selective antibiotics were used as follows: w/o plasmid: none; pCM160 or pMis1_1B: kanamycin; pMis1_1B + pCM80: kanamycin + tetracycline. Three independent biological replicates were measured. Colored areas indicate the standard deviation (SD).

**Figure A1:**
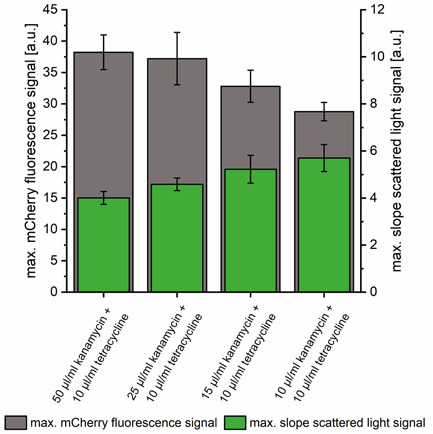
Effect of variation of kanamycin concentration on the growth of *Methylorubrum extorquens* AM1 pCM80/pMis1_1B_P_mxaF__*mCherry* cotransformants. The maximal mCherry fluorescence signal is a measure of pMis1_1B_P_mxaF__*mCherry* abundance, while the maximal slope of the scattered light signal is an indicator of growth rate. Three independent biological replicates were measured; standard deviations are indicated by error bars.

For testing the compatibility of pMis1_1B with the pCM system, *M. extorquens* AM1 was transformed with pMis1_1B and pCM80 simultaneously. The cotransformation did lower the transformation efficiency by 2‐fold compared to transformation of pMis1_1B alone (Figure 2a). Furthermore, colonies of cotransformants were only clearly visible after 8 days of growth, indicating an increased metabolic burden caused by the presence of both plasmids. Detailed growth monitoring in liquid growth medium confirmed these results: While *M. extorquens* AM1 harboring pMis1_1B already showed a reduced growth rate compared to the pCM160‐containing strain or the plasmid‐free strain, cotransformation with both plasmids strongly reduced the growth rate (Figure 2b). Plasmid‐induced reduction of growth rates under methylotrophic growth conditions due to metabolite limitations have already been described for *M. extorquens* AM1 (Kiefer et al., 2009). We, therefore, tested increased amounts of several media components but were unable to identify any medium‐related limitation (data not shown). Only a reduction in the kanamycin concentration resulted in an increased growth rate, but most probably reduced plasmid maintenance stability or copy number, as revealed by fluorescence quantification experiments using an mCherry reporter derivative of pMis1_1B (Figure A1). Mutations in the *rep*‐gene region were already successfully used to increase the copy number of pBBR‐based broad host range plasmids and the growth rate of corresponding transformants (Mi et al., 2016; Tao et al., 2005). A more directed screening of *rep* gene expression level variants might identify a more suitable expression level associated with less plasmid burden.

### 3.3 Characterization of pMis1_1B as pCM coexpression vector

**Figure 3:**
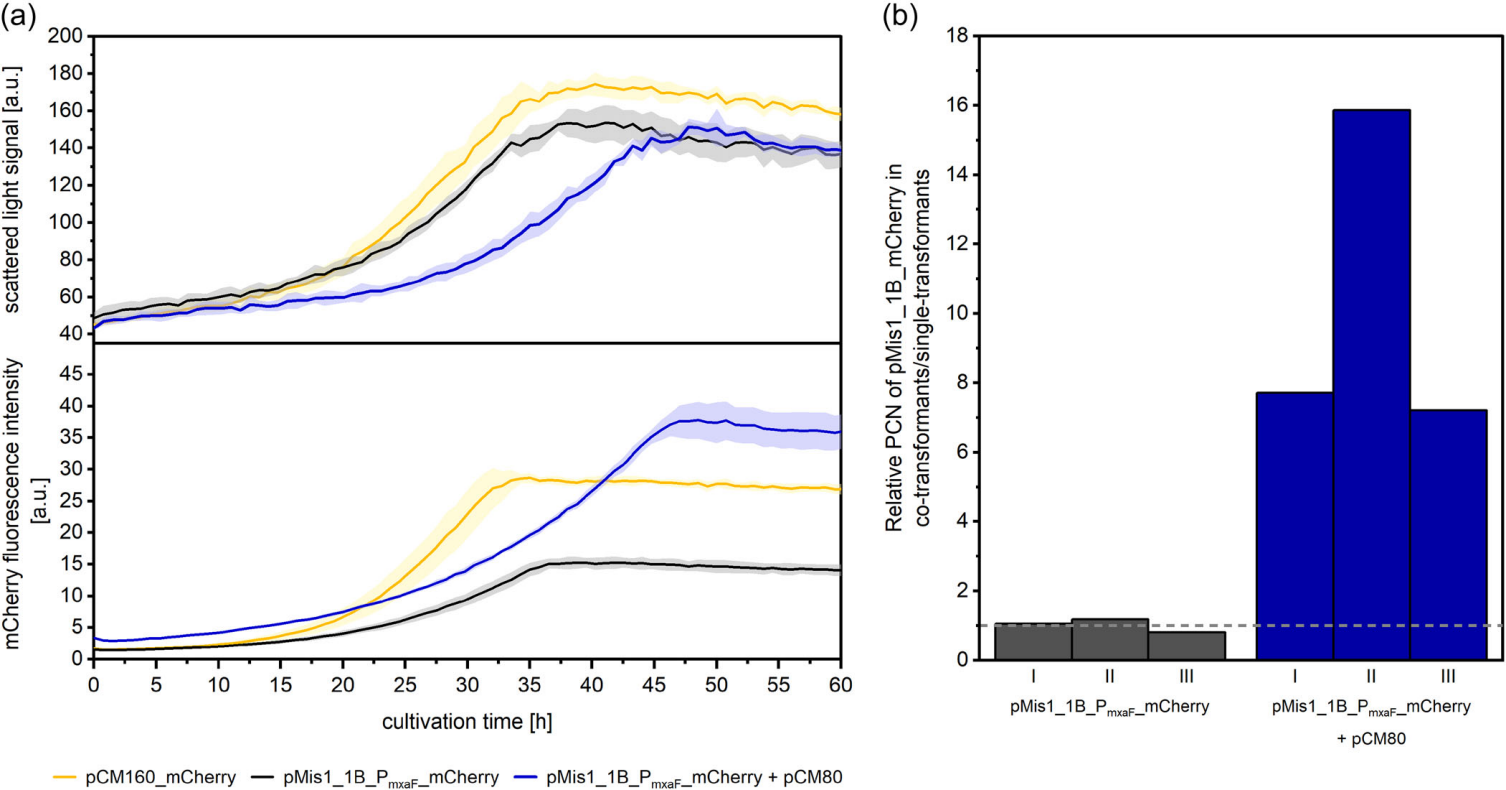
Investigation of phenotypes for *Methylorubrum extorquens* AM1 strains carrying pCM160 and pMis1_1B_P_mxaF__*mCherry*. (a) Growth and mCherry fluorescence signal of *M. extorquens* AM1 containing pCM160, pMis1_1B_P_mxaF__*mCherry*, or both plasmids in combination. Three independent biological replicates were investigated in a microbioreactor system. Colored areas indicate the standard deviation (SD). (b) Ratio of relative PCN of pMis1_1B_P_mxaF__*mCherry* in *M. extorquens* AM1 + pMis1_1B_P_mxaF__*mCherry* + pCM80 to the same plasmid in *M. extorquens* AM1 + pMis1_1B_P_mxaF__*mCherry*. The chromosomal housekeeping gene was used as a reference. The mean value of relative pMis1_1B_P_mxaF_ _*mCherry* PCN of the control strain (= 1) is shown by a dashed line. For each strain, three independent transformants (I–III) were measured.

**Figure A2:**
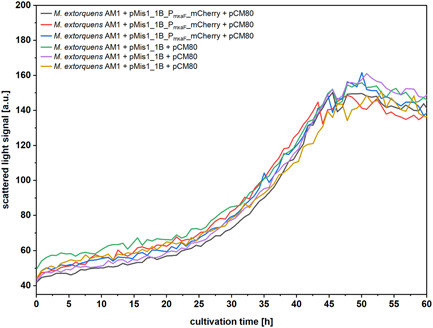
Comparison of scattered light measurements of *Methylorubrum extorquens* AM1 cotransformants containing pCM80 combined with either pMis1_1B or pMis1_1B_P_mxaF__*mCherry*. Measurements of biological triplicates are shown.

Despite the lower growth rates of the cotransformants, pMis1_1B is nevertheless a promising candidate for simultaneous gene expression from two plasmids in *M. extorquens* AM1. To investigate the plasmid stability and general expression levels from pMis1_1B, we used pMis1_P_mxaF__*mCherry* containing the reporter gene under the control of the strong native constitutive promotor P_mxaF_. Expression of *mCherry* did not change the growth behavior of the respective strains (Figure A2). We investigated the *mCherry* expression levels from respective pCM and pMis1_1B constructs in strains with one or two plasmids, respectively. The previously described growth defect of the cotransformants was also visible in this experiment (Figure 3a). However, there was a clear synergistic effect in terms of expression: The mCherry signal originating from pMis1_1B_P_mxaF__*mCherry* was substantially increased if pCM80 was additionally present. The maximal mCherry fluorescence signal of the cotransformants even exceeded the values from pCM160_*mCherry* single transformants. One explanation for this finding could be an increased PCN. We, therefore, determined the relative PCN of pMis1_1B_*mCherry* for strains containing two plasmids and strains containing only a single plasmid by real‐time PCR. Since the efficiency of the used primer pairs varied from 1.73 to 1.96, the E‐corrected Pfaffl‐method was used (Pfaffl, 2004). Although the values determined for each of the three replicate strains containing two plasmids showed a strong variation, a clear increase in PCN was detectable when both plasmids are present in *M. extorquens* AM1 (Figure 3b). Thus, the presence of pCM80 led to a strong increase in the pMis1_1B copy number.

## 4 CONCLUSIONS

The pBBR1 derivative pMis1_1B, which we characterized in this study represents a novel plasmid for *M. extorquens*, which is compatible with the widely used pCM system. Even though the growth rate of *M. extorquens* AM1 was affected when both plasmids were present in the cells, pMis1_1B could be a powerful tool for certain applications. For example, it provides enormous facilitation for the combinatorial testing of multiple enzymes during the development of synthetic pathways. Moreover, a new plasmid system that is not as stably maintained as pCM could be useful for applications where an expression plasmid is only needed transitionally as for the expression of Cre‐recombinase in a Cre/*loxP* recombination system or for CRISPR gene editing. With pMis1_1B, a new useful tool has been added to the expanding genetic toolbox for *M. extorquens*.

## AUTHOR CONTRIBUTIONS

**Laura Pöschel**: Conceptualization (equal); investigation (equal); methodology (equal); visualization (lead); writing–original draft (lead); writing–review and editing (equal); **Elisabeth Gehr**: Conceptualization (equal), investigation (equal), methodology (equal); writing–original draft (supporting); **Markus Buchhaupt**: Conceptualization (equal); methodology (equal); funding acquisition (lead); project administration (lead); writing–original draft (supporting); writing–review and editing (equal).

## CONFLICT OF INTEREST

None declared.

## ETHICS STATEMENT

None required.

## Data Availability

The plasmid sequence of pMis1_1B is available in the NCBI GenBank under accession number OP441404: https://www.ncbi.nlm.nih.gov/nuccore/OP441404. The plasmid itself has been deposited to Addgene. All other data generated or analyzed during this study are included in this published article.
